# Selective AKT Inhibition by MK-2206 Represses Colorectal Cancer-Initiating Stem Cells

**DOI:** 10.1245/s10434-016-5218-z

**Published:** 2016-04-08

**Authors:** Patrizia Malkomes, Ilaria Lunger, Alexander Luetticke, Elsie Oppermann, Nadine Haetscher, Hubert Serve, Katharina Holzer, Wolf Otto Bechstein, Michael A. Rieger

**Affiliations:** 1 Department of General Surgery, Goethe University Hospital Frankfurt, Frankfurt, Germany; 2 LOEWE Center for Cell and Gene Therapy Frankfurt and Department of Medicine, Hematology/Oncology, Goethe University Hospital Frankfurt, Frankfurt, Germany; 3 German Cancer Consortium (DKTK), Heidelberg, Germany; 4 German Cancer Research Center (DKFZ), Heidelberg, Germany

## Abstract

**Background:**

Colorectal cancer (CRC) is a leading cause of cancer-related death worldwide. Growing evidence indicates that tumor-initiating cells (TICs) are responsible for tumor growth and progression. Conventional chemotherapeutics do not sufficiently eliminate TICs, leading to tumor relapse. We aimed to gain insight into TIC biology by comparing the transcriptome of primary TIC cultures and their normal stem cell counterparts to uncover expression differences.

**Methods:**

We established colonosphere cultures derived from the resection of paired specimens of primary tumor and normal mucosa in patients with CRC. These colonospheres, enriched for TICs, were used for differential transcriptome analyses to detect new targets for a TIC-directed therapy. Effects of target inhibition on CRC cells were studied in vitro and in vivo.

**Results:**

Pathway analysis of the regulated genes showed enrichment of genes central to PI3K/AKT and Wnt-signaling. We identified CD133 as a marker for a more aggressive CRC subpopulation enriched with TICs in SW480 CRC cells in an in vivo cancer model. Treatment of CRC cells with the selective AKT inhibitor MK-2206 caused a decrease in cell proliferation, particularly in the TIC fraction, resulting in a significant reduction of the stemness capacity to form colonospheres in vitro and to initiate tumor formation in vivo. Consequently, MK-2206 treatment of mice with established xenograft tumors exhibited a significant deceleration of tumor progression. Primary patient-derived tumorsphere growth was significantly inhibited by MK-2206.

**Conclusion:**

This study reveals that AKT signaling is critical for TIC proliferation and can be efficiently targeted by MK-2206 representing a preclinical therapeutic strategy to repress colorectal TICs.

**Electronic supplementary material:**

The online version of this article (doi:10.1245/s10434-016-5218-z) contains supplementary material, which is available to authorized users.

Colorectal cancer (CRC) is the second most common cancer worldwide.^1^ Although numerous improvements in treatment modalities have been achieved, approximately 40 % of patients will still die from recurrent or metastatic disease within 5 years.^2^ Consequently, conventional therapeutic strategies are unable to eliminate all cancer cells.

CRC is a stem-cell-driven malignancy in which only a small population of cells, simplified as tumor-initiating cells (TICs), are able to initiate and sustain tumor growth.^3^ TICs are undifferentiated tumor cells with the exclusive ability to self-renew and to generate the cellular heterogeneity of a tumor. TICs are more resistant to conventional anticancer therapy and therefore may be the main cause of treatment escape and tumor relapse.^4^–^6^

Initially, the TIC population in CRC was identified by the presence of the surface marker CD133, which showed an increased tumorigenic potential in xenografts of immunodeficient mice.^7^ Despite the description of some surface markers, only an insufficient purity of TICs can be achieved so far and their biology remains undefined.^8^ Hence, identifying the regulatory mechanisms and signaling pathways involved in TICs, and developing targeted therapy, might raise promising strategies in the treatment of CRC.

Emerging data revealed PI3K/AKT/mTOR signaling implicated in the progression of CRC and that components of the mTOR pathway were overexpressed in CRC.^9^ In recent studies, a new oral-specific AKT1/2/3 inhibitor, MK-2206, provided in vitro and in vivo antitumor activity as a single agent, as well as enhanced activity in combination with conventional chemotherapeutics.^10^–^13^ In addition, MK-2206 has been shown to be safe in humans, with early evidence of antitumor activity in clinical trials.^14^,^15^

The present study aimed to determine the phenotypic and molecular differences between colonic TICs and their normal colon stem cell counterparts. Transcriptome analyses revealed that genes involved in AKT signaling are enriched in the TIC cultures. Functional testing implicated the selective AKT inhibitor MK-2206 as a potential therapeutic for TIC-directed therapy in CRC.

## Methods

### Patient Material

Human colon cancer and adjacent normal mucosa tissue were obtained after surgical resection and characterization by a pathologist. Tissue collection was approved by the Ethics Committee of the University Hospital Frankfurt, and after written consent had been received from all patients involved in the study. Solid tissues were minced and dissociated with 200 U/ml Collagenase type III, 100 U/ml Dispase, and 100 U/ml DNase I (all Worthingtorn, USA) in HBSS for 60–90 min at 37 °C. Every 30 min the cell suspension was subjected to MACS tissue dissociator for 40 s. Cells were filtered through sterile 70 µm nylon mesh [Becton Dickinson (BD), Heidelberg, Germany], and contaminated red blood cells were removed by osmotic lysis.

### Sphere Formation Assay

Isolated cells were suspended in serum-free DMEM/F12 (Gibco, Germany) supplemented with 20 ng/ml epidermal growth factor and fibroblast growth factor, 2 % N2 supplement (Life Technologies, Germany), 20 mmol/l HEPES, and 50 U/ml penicillin/streptomycin at a density of 50,000 cells (tumor) and 100,000 cells (normal) per well in ultra-low-attachment 24-well plates (Corning, Germany), as described by Kreso and O’Brien.^16^ Plates were scored microscopically after 7 and 14 days.

### Microarray Analysis

Expression analysis was performed using Genechip Human Exon 1.0 ST. Array (Affymetrix, Santa Clara, CA, USA). RNA was extracted from 14-day tumorspheres and corresponding colonospheres from normal tissue using an RNeasy Midi kit according to the manufacturer’s instructions. RNA quantity and quality were assessed using Nanovue (GE Life Sciences, USA) and 2100 Bioanalyzer (Agilent, USA), respectively. Only samples with a high RNA integrity number (RIN: 8–10) were used for the profiling. Genes with a twofold cut-off were then further subdivided into functional categories and pathways with the bioinformatics analysis resource DAVID (Database for Annotation, Visualization and Integrated Discovery) of the Laboratory of Immunopathogenesis and Bioinformatics.

### Cell Culture

Human CRC cell lines SW480 and hematocrit (HCT)-116 were cultured in McCoy’s 5a and CX-1 in MEM-Earls containing 10 % FCS, 200 mM HEPES, 2 mmol l-glutamine, 50 units/ml penicillin/streptomycin in 37 °C humidified atmosphere with 5 % CO_2_. All cell lines were purchased from CLS Cell Lines Service GmbH, Eppelheim, Germany.

### In Vitro Drug Treatment

MK-2206 (Selleckchem, Munich, Germany) and 5-fluorouracil (Sigma-Aldrich, Munich, Germany) were dissolved in dimethyl sulfoxide (DMSO) and stored at −20 °C. 5000 SW480, HCT-116 and CX-1 cells per well in 96-well plates were cultured with or without 5-fluorouracil (10 µM) and combination treatment with or without MK2206 (5 µM) for 24–72 h. Proliferation was assessed using a 3-(4,5-dimethylthiazol-2yl)-2,5-diphenyltetrazolium bromide (MTT) assay according to the manufacturer’s instructions in pentaplicate. For apoptosis and cell cycle assays, 25,000 cells per well were cultured in 24-well plates under the same treatment as previously described. Apoptosis was determined 24 and 72 h after treatment using AnnexinV/7AAD staining (BD, Germany), according to the manufacturer’s instructions, after cells were stained with anti CD133-PE. To determine the cell cycle distribution, 48 and 72 h after treatment cells were pulsed with bromodeoxyuridine (BrdU) for 8 h, stained with anti-CD133-PE followed by fixation/permeabilization and anti-BrdU and DNA content (7AAD) staining using an FITC BrdU flow kit (BD, Germany), according to the manufacturer’s instructions. The cells were analyzed on an FACSCanto II (BD, Germany).

The effect of treatment on tumorsphere formation was investigated by sphere formation assay as described above.

### Flow Cytometry and Cell Sorting

SW480 cells were detached with Accutase™ (Sigma-Aldrich, Germany) and incubated with a PE-conjugated antibody to human CD133 (clone AC133/1 Miltenyi Biotec, Bergisch Gladbach, Germany) or a mouse isotype control. Stained cells were analyzed via a FACSCanto or sorted via a FACSAria (BD, Germany).

### In Vivo Xenograft Experiments

All animal experiments were approved by the local authorities. NOD.Cg-Prkdc^*scid*^I12rg^*tm1Wjl*^/SzJ (NSG) or NOD.CB17-*Prkdc*^*scid*^/J (non-obese diabetic/severe combined immunodeficiency [NOD/SCID]) mice (Jackson Laboratory, USA) were used at 6–8 weeks of age. Fluorescence-activated cell sorting (FACS)-sorted SW480 cells (CD133^high^, CD133^+^, CD133^−^) were resuspended 1:1 in Matrigel (BD, Germany) and serum-free medium, and were injected subcutaneously into the flank at 5 × 10^4^ cells per mouse. For the in vivo treatment, MK-2206 (100 mg/kg) was administered orally three times a week when tumor size reached 0.2–0.3 cm in diameter. Tumor growth was measured twice weekly using a caliper, and mice were sacrificed when tumor size reached a diameter of 1 cm.

### Western Blot

Heat-denatured protein samples were separated using SDS-PAGE and transferred to a PVDF membrane (GE Healthcare, Germany) by electroblotting (Bio-Rad, Germany). Blots were blocked with 10 % milk for 1 h and incubated with primary antibodies against total Akt, phosphoAkt, or phosphoRPS6 (all Cell Signaling Technology, Danvers, MA, USA) overnight at 4 °C. Proteins were detected with peroxidase-goat anti-mouse antibody and ECL (GE Healthcare, Germany) on a Fusion FX7 imaging system (Vilber Lourmat, France). β-Actin (Sigma-Aldrich, Germany) was used as the internal loading control.

### Statistical Analysis

Statistical analyses were performed using GraphPad Prism 6 (GraphPad Software, Inc., La Jolla, CA, USA). The microarray data were statistically analyzed using a *t* test, by ATLAS Biolabs GmbH.

## Results

Gene expression revealed upregulated AKT, P53, and Wnt signaling in primary tumorspheres.

**Fig. 1:**
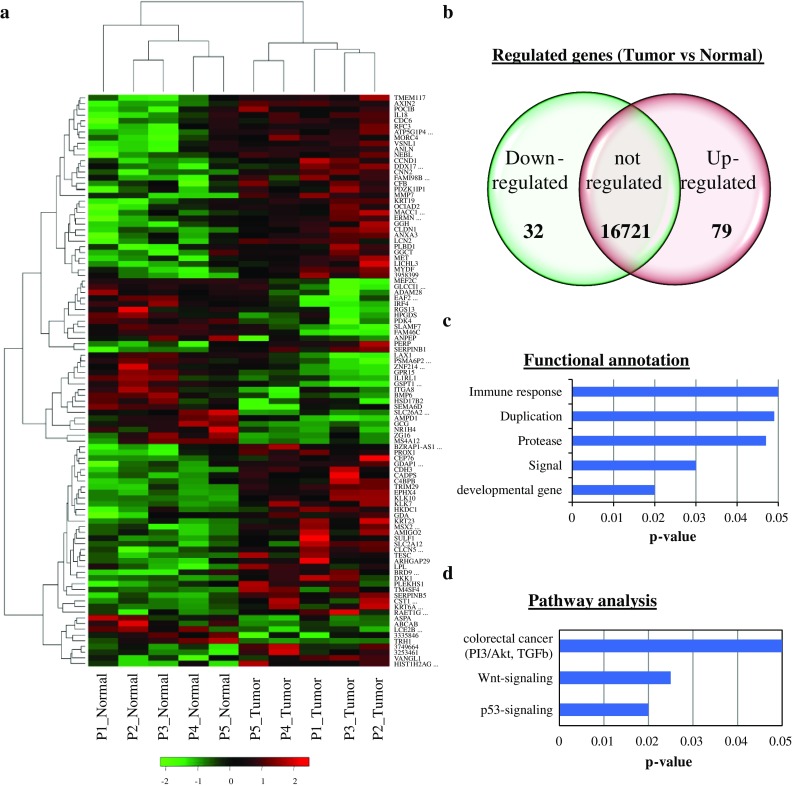
Transcriptome profiling revealed upregulated AKT, P53, and Wnt signaling pathways in CRC spheres. Exon-array expression analyses of matched CRC tumorspheres/corresponding normal colonospheres from five different patients. **a** Heat map showing a two-dimensional clustering of the top 100 regulated genes. **b** Venn diagram of regulated genes, tumor versus normal (*p* < 0.05, fold change >2). **c** DAVID functional annotation. **d** Pathway analysis of differentially expressed genes in tumorspheres. The top functions (**c**) and main canonical pathways (**d**) with their corresponding *p* values are shown. *CRC* colorectal cancer

First, we aimed to determine changes in gene expression patterns and regulated pathways in TICs. Therefore, we compared the transcriptome of five paired samples consisting of tumorsphere-initiating cells and corresponding non-malignant colonosphere-forming cells from five different CRC patients. The spheres were derived from freshly resected cancer tissue and corresponding normal colon mucosa after a 14-day culture. FACS analysis showed a strong enrichment of CD133^+^ cells in spheres (82.7  ± 7.5 %) in comparison to monolayer culture (16.9 ± 11 %; *n* = 6, data not shown). The differential expression analysis revealed 79 induced and 32 repressed genes in tumorspheres in comparison to normal colonospheres (Fig. 1a, b, and Electronic Supplementary Table 1). Well-described genes that are involved in CRC progression were upregulated in tumorspheres, such as *MET* and *MACC1*.^17^ Next, we included the differentially expressed genes in a functional annotation analysis using the DAVID algorithm. When classified according to function, genes regulating ‘immune response’, ‘duplication’ and ‘protease’ were most frequently identified (Fig. 1c). Importantly, well-described pathways associated with CRC progression, such as ‘Wnt signaling’ (DKK, Axin, CCND1), ‘P53 signaling’ (PERP, SERPINB5), and ‘colorectal cancer’, including AKT (CCND1, TSC, Axin, MET) and transforming growth factor (TGF)-β signaling, were also overrepresented in tumorspheres (Fig. 1d) and were thus potential targets for a TIC-directed therapy.

High CD133 expression demarcates an aggressive TIC subset in colorectal cancer SW480 cells.

**Fig. 2:**
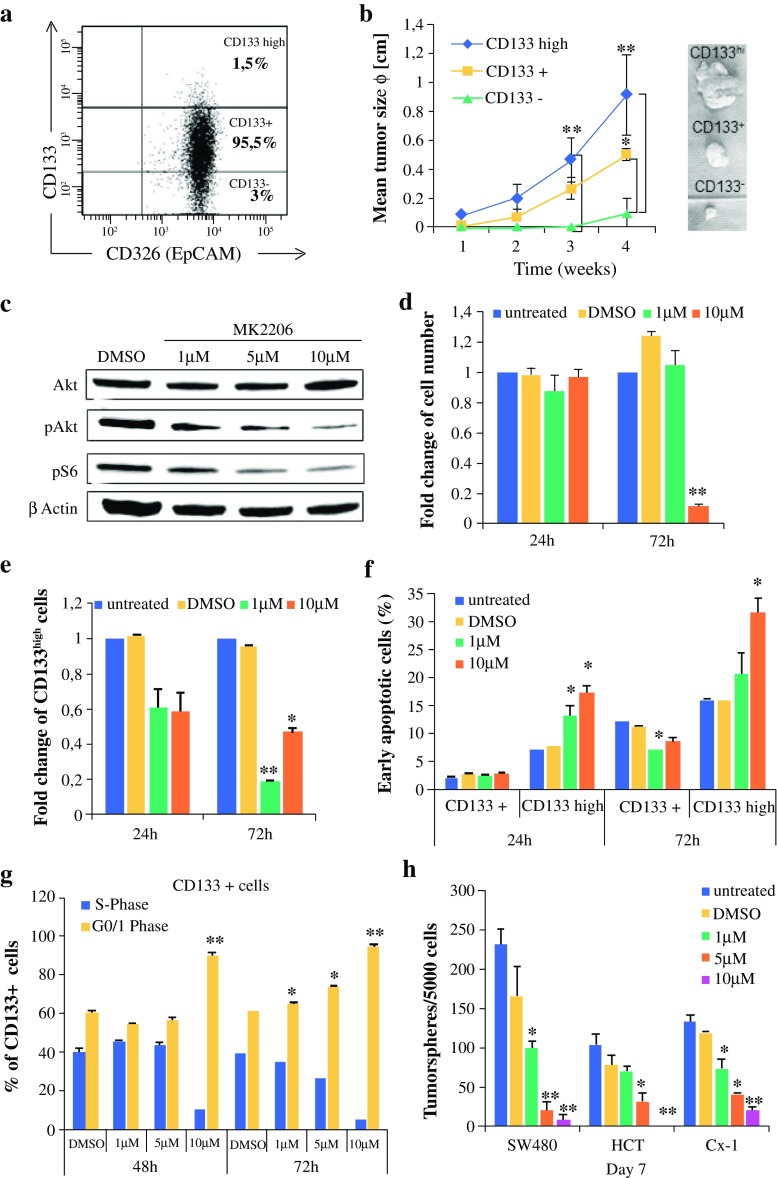
Selective AKT inhibition by MK-2206 diminishes TIC activity and survival in vitro. **a** Three subpopulations in SW480 cells based on CD133 expression. Representative FACS blot. **b** Tumor growth of 50,000 prospectively isolated CD133 subpopulations injected subcutaneously in NSG (*n* = 4). **c**–**g** SW480 cells were treated with 1, 5, or 10 µM MK-2206 for the indicated times. **c** Western blot analysis after 72 h of treatment. **d** Cell proliferation (MTT assay). **e** Fold change of CD133^high^-expressing cells detected via FACS. **f** Early apoptotic cells (AnnexinV^+^/7-AAD^−^) in CD133 subpopulations determined via FACS. (**g**) Cell cycle phase distribution in CD133^+^ bulk cells detected via BrdU incorporation. **h** Tumorsphere formation in CRC cell lines. Data are expressed as mean and standard deviation. *p < 0.05; **p < 0.01 compared with DMSO. *TIC* tumor-initiating cell, *FACS* fluorescence-activated cell sorting, *DMSO* dimethyl sulfoxide, *MTT* 3-(4,5-dimethylthiazol-2yl)-2,5-diphenyltetrazolium bromide, *BrdU* bromodeoxyuridine, *CRC* colorectal cancer, *HCT* hematocrit

To further investigate the role of TIC biology, we employed SW480 CRC cells, given that they are genetically similar to most sporadic CRCs. FACS analysis of the CRC cell line SW480 revealed a differential surface expression of the described TIC marker CD133^7^,^18^ that allowed us to prospectively separate three subsets of cells, CD133^−^ (3 %), CD133^+^ (95.5 %), and CD133^high^ (1.5 %) cells (Fig. 2a).

To confirm that CD133 is a marker of tumor initiation in SW480 cells, all three subpopulations were prospectively isolated via FACS based on their CD133 expression, and subcutaneously inoculated in NSG mice to monitor tumor formation. Indeed, CD133^high^ cells caused the most robust and rapid establishment of tumors, and the tumors progressed faster than tumors from the CD133^+^ or CD133^−^ populations (Fig. 2b). These results identify an aggressive TIC population in SW480 cells.

### Selective AKT Inhibition Diminishes Colonic Tumor-Initiating Cells In Vitro

Next, we assessed the effect of AKT inhibition on TIC activity and survival. To this aim, SW480 cells were treated with MK-2206, a selective AKT1/2/3 inhibitor, for 72 h. MK-2206 treatment for 24 h inhibited AKT kinase activity (Fig. 2c). To evaluate the consequences of AKT inhibition on tumor cell growth, CRC cells were treated with MK-2206, and their cell expansion was analyzed. At 10 µM, MK-2206 significantly reduced cell proliferation after 72 h of treatment (Fig. 2d). Next, we were able to demonstrate that MK-2206 significantly decreased the amount of the CD133^high^ subset after 72 h of treatment (Fig. 2e), indicating that MK-2206 treatment specifically eradicated the TIC subset in CRC cells.

To further investigate whether the effects induced by MK-2206 were evoked by apoptosis induction or cell cycle inhibition, we determined AnnexinV/7AAD staining and BrdU incorporation in SW480 cells treated for 72 h via FACS, to simultaneously discriminate the various CD133-expressing cell subsets. While MK-2206 did not induce apoptosis within 72 h in the CD133^+^ bulk population of cancer cells, we observed enhanced apoptosis in the TIC-enriched fraction after 24 and 72 h of treatment (Fig. 2f). In addition to a selective effect on apoptosis in the TIC fraction, we determined a dose-dependent cell cycle G1 arrest in the bulk of SW480 cells treated with MK-2206, as assayed by the incorporation of BrdU during the S-phase of the cell cycle (Fig. 2g). To ensure that our findings were not restricted to one cell line, we performed the tumorsphere formation assay in two additional CRC cell lines to functionally confirm the effect of AKT inhibition on TIC activity. After 7 days in culture, the number of tumorspheres were significantly diminished by MK-2206, even at low concentrations in all three cell lines (Fig. 2h). Taken together, AKT inhibition with MK-2206 reduces CRC cell proliferation and directly inhibits TIC survival and function in vitro.

### MK-2206 Potentiates the Effect of 5-Fluorouracil Chemotherapy

**Fig. 3:**
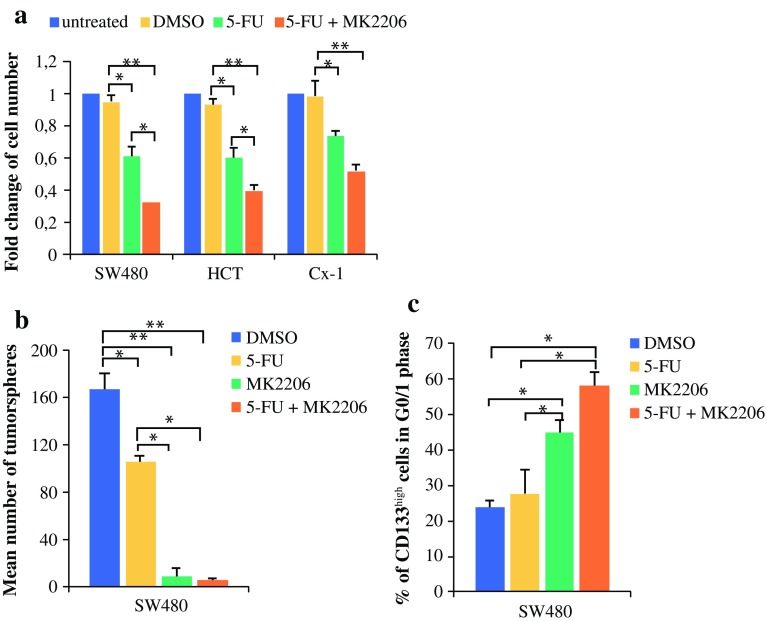
Synergistic antitumoral effects of 5-fluorouracil and MK-2206 in CRC. **a**–**c** SW480, HCT and Cx-1 cells were treated with either 5 µM MK-2206, 10 µM 5-fluorouracil, or in combination. **a** Cell proliferation was determined after 48 h (MTT assay). **b** SW480 tumorspheres scored after 14 days of treatment. **c** Percentage of CD133^high^-SW480 cells in G1 phase after 72 h of treatment detected via an 8 h BrdU pulse. Data are expressed as mean and standard deviation. *p < 0.05; **p < 0.01 compared with DMSO. *CRC* colorectal cancer, *DMSO* dimethyl sulfoxide, *HCT* hematocrit, *5*-*FU* 5-fluorouracil, *MTT* 3-(4,5-dimethylthiazol-2yl)-2,5-diphenyltetrazolium bromide, *BrdU* bromodeoxyuridine

To elucidate the antitumoral effect of MK-2206, we analyzed its impact on CRC cell proliferation and tumorsphere formation when co-treated with the standard chemotherapeutic drug 5-fluorouracil in CRC treatment. The combined treatment of MK-2206 and 5-fluorouracil caused a significant inhibition of cell proliferation compared with 5-fluorouracil alone, indicating a synergistic anticancer effect of both drugs on CRC cells (Fig. 3a).

We then investigated whether the combination therapy would be effective in inhibiting TIC proliferation. MK-2206 alone was highly effective in inhibiting tumorsphere formation in SW480 cells, while a combined treatment with 5-fluorouracil did not further increase this effect (Fig. 3b). Furthermore, a single treatment with 5-fluorouracil did not cause cell cycle arrest in TICs, whereas MK-2206 alone and combined treatment with 5-fluorouracil significantly induced G1 cell cycle arrest in TIC-enriched fraction after 72 h (Fig. 3c). Although MK-2206 showed a synergistic antitumor effect when combined with 5-fluorouracil, MK-2206 alone was highly effective in suppressing colon TIC activity.

### Selective AKT Inhibition Reduces Tumor Initiation and Growth In Vivo and Patient-Derived Tumorsphere Formation

**Fig. 4:**
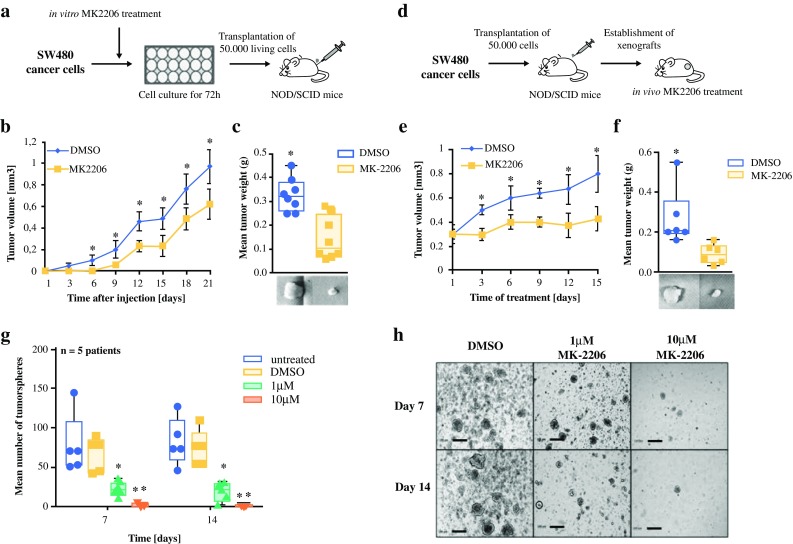
AKT inhibition by MK-2206 reduces tumor initiation and growth in xenografts and tumorsphere formation in primary patient material. **a** SW480 cells were pretreated with 10 µM MK2206 for 72 h in vitro and, subsequently, 50,000 living cells were injected subcutaneously (*n* = 8). **b** Tumor volumes over time. **c** Tumor weight after 4 weeks. **d** Equal numbers of SW480 cells were injected subcutaneously. Once the tumor reached 25 mm^3^, mice were randomized into two groups and treated with MK-2206 (100 mg/kg, three times/week) or DMSO (control) [*n* = 6]. **e** Tumor volumes under treatment over time. **f** Tumor weight after 3 weeks of treatment. **g** Tumor cells from primary CRC patient material were treated with DMSO or MK-2206, and sphere formation was determined (*n* = 5 CRC patients). **h** Representative images of sphere formation. *Scale bar* 200 µm. Data are expressed as mean and standard deviation. *p < 0.05; **p < 0.01 compared with DMSO. *DMSO* dimethyl sulfoxide, *CRC* colorectal cancer, *NOD* non-obese diabetic, *SCID* severe combined immunodeficiency

Since TIC activity can only be reliably determined by its capacity to initiate tumor growth in vivo, we assessed the potency of MK-2206 in a xenograft transplantation model. Therefore, we pretreated SW480 cells with MK-2206 or DMSO for 72 h in culture before we subcutaneously injected equal numbers of living cells into NOD/SCID mice (Fig. 4a). The treatment with MK-2206 significantly delayed tumor formation and significantly reduced tumor growth (mean tumor weight 0.32 g vs. 0.12 g; *p* < 0.05) (Fig. 4b, c) induced by the remaining cells after the pretreatment, suggesting that MK-2206 selectively eliminates TICs.

To further demonstrate the anticancer effects of MK-2206 in vivo, NOD/SCID mice with established SW480 tumor cell xenografts were treated with MK-2206 or DMSO (control) (Fig. 4d). While the DMSO-treated mice showed a successive increase in tumor growth, MK-2206 treatment resulted in an almost complete stagnation of tumor size, leading to small tumors that were as equal in size as before the start of treatment.(Fig. 4e, f).

In order to translate our results on MK-2206 activity against TICs to primary CRC patients, tumorsphere cultures derived from freshly isolated patient CRC tissue were treated with MK-2206. After 7 and 14 days of treatment, the number and diameter of formed tumorspheres were significantly reduced by MK-2206 compared with the DMSO control (Fig. 4g, h). These results suggest that AKT inhibition effectively impacts on TIC activity and serves as a treatment strategy against colorectal TICs.

## Discussion

In this study, we report the effectiveness of selective AKT inhibition by MK-2206 on diminishing the tumorigenic potential of CRC-intiating cells. Although already highly effective alone, MK-2206 showed synergistic anticancer effects on CRC cell proliferation with conventional chemotherapeutics such as 5-fluorouracil. Of note, the effects of AKT inhibition on TIC activity could be determined in both mismatch repair proficient (SW480) and deficient (HCT-116) CRC cells. Importantly, MK-2206 reduced tumorsphere formation in cancer cells derived from primary patient specimens. Similar to recent results, treatment with MK-2206 generated a significant deceleration in tumor progression in vivo.^19^

mTOR/AKT signaling is essential in the progression of CRC, and mTOR inhibition leads to a reduction of CRC cell growth.^8^,^9^,^20^ Cai et al. identified mTOR high expression to be an independent risk factor for the prognosis of CRC patients.^21^ An activation of mTOR signaling has been detected in colon TICs.^22^,^23^ In concordance with these reports, the present study detected an increased expression of components of the AKT/mTOR signaling in colon TICs.

Few studies have investigated the effects of mTOR inhibitors on colon TICs. In one study, treatment with rapamycin and PP242 diminished sphere formation as well as aldehyde dehydrogenase activity in vitro.^21^ However, the authors did not perform sufficient functional experiments to investigate the effect of mTOR inhibitors on the tumorigenic potential of colon TICs. A second study proposed the mTOR kinase inhibitor Torin-1 as a drug candidate for CRC therapy as it inhibited survival of colon TICs in vitro and slowed tumor progression in vivo.^22^ Similarly, Todaro et al. showed, that PI3K-inhibition by BKM-120 reduced metastatic growth of CRC cells.^23^ Nevertheless, no further studies have been reported to elucidate the effectiveness of Torin-1 in cancer or its safety in patients. Furthermore, the authors did not assess the effects of Torin-1 or BKM-120 on the tumor initiation ability of TICs.

## Conclusions

We achieved a significant reduction of the tumor growth originating from TICs, both in vitro and in vivo, by selective AKT inhibition with MK-2206. Given that AKT is both an upstream activator of the mTOR complex 1 and a downstream effector of the mTOR complex^2^,^24^ and its overexpression is associated with resistance to chemotherapy,^25^ it represents a critical target of this signaling cascade. Therefore, selective AKT inhibition may be superior to mTOR inhibitors in the treatment of CRC, and represents a promising agent to prevent tumor relapse by eliminating the TIC subset. Our data strongly encourage further clinical testing of MK-2206 either alone or in combination with conventional chemotherapeutics in CRC patients.

## Electronic Supplementary Material

Below is the link to the electronic supplementary material.

Supplementary material 1 (XLSX 18 kb)
